# Supplementary material for: Storing and managing water for the environment is more efficient than mimicking natural flows
Source: Nat Commun. 2024 Jun 27;15:5462. doi: 10.1038/s41467-024-49770-4 (PMC11211385; doi:10.1038/s41467-024-49770-4)
Supplement: Supplementary file 1 — Supplementary Information [file 41467_2024_49770_MOESM1_ESM.pdf]

Supplemental Figures

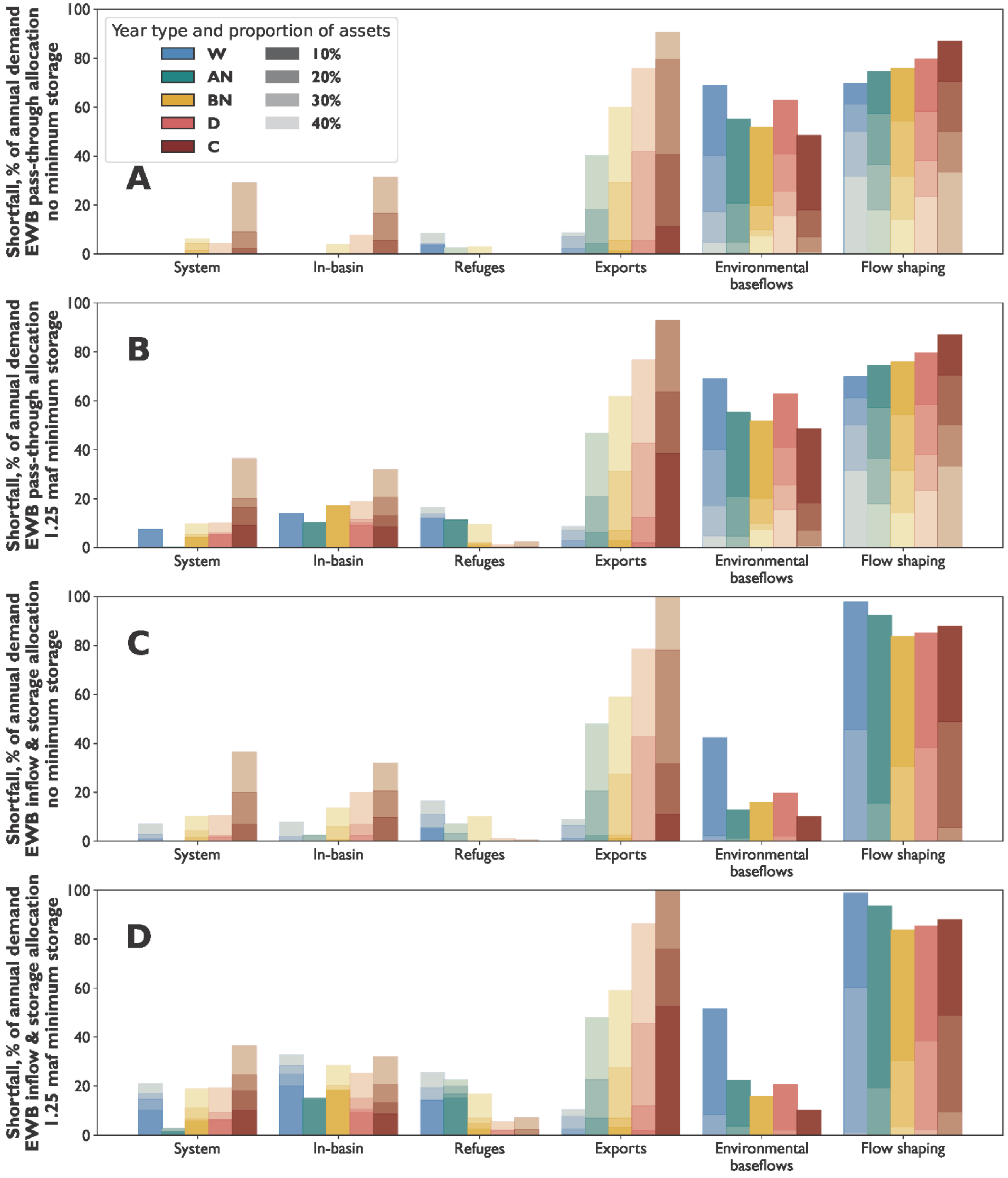

Supplemental Figure 1. Average annual shortfalls across 26 years of hydrologic conditions for each water demand by water year type. Panel A) percentage of pass-through with no minimum reservoir storage, B) percentage of inflow with 1.54 Bm^3 minimum reservoir storage, C) percentage of inflow and a percentage of reservoir storage capacity with no minimum reservoir storage, and D) percentage of inflow and a percentage of reservoir storage capacity with 1.54 Bm^3 minimum reservoir storage. W = wet years, AN = above normal years, BN = below normal years, D = dry years, and C = critically dry years using the Sacramento River Index.

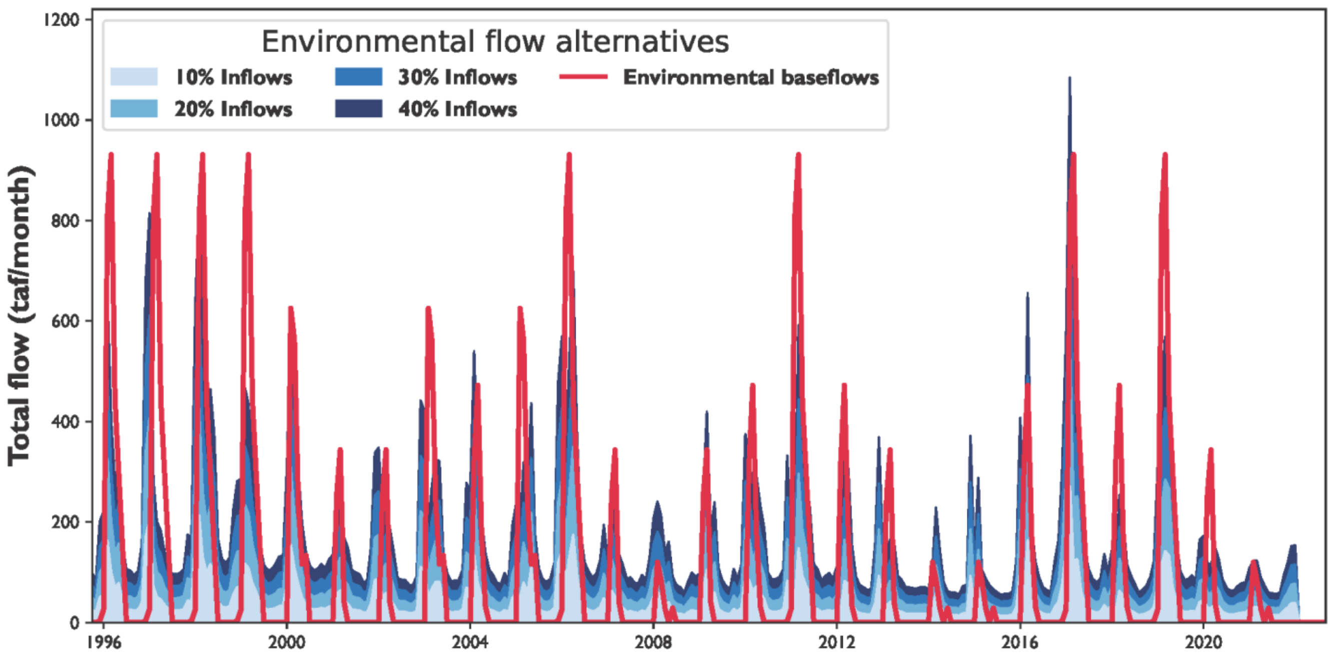

Supplemental Figure 2. Proportional environmental water inflow allocations from the experimental reservoir, compared to historical environmental baseflows. Inflow proportions are derived from Shasta Reservoir inflow data (Shasta Dam USBR station).

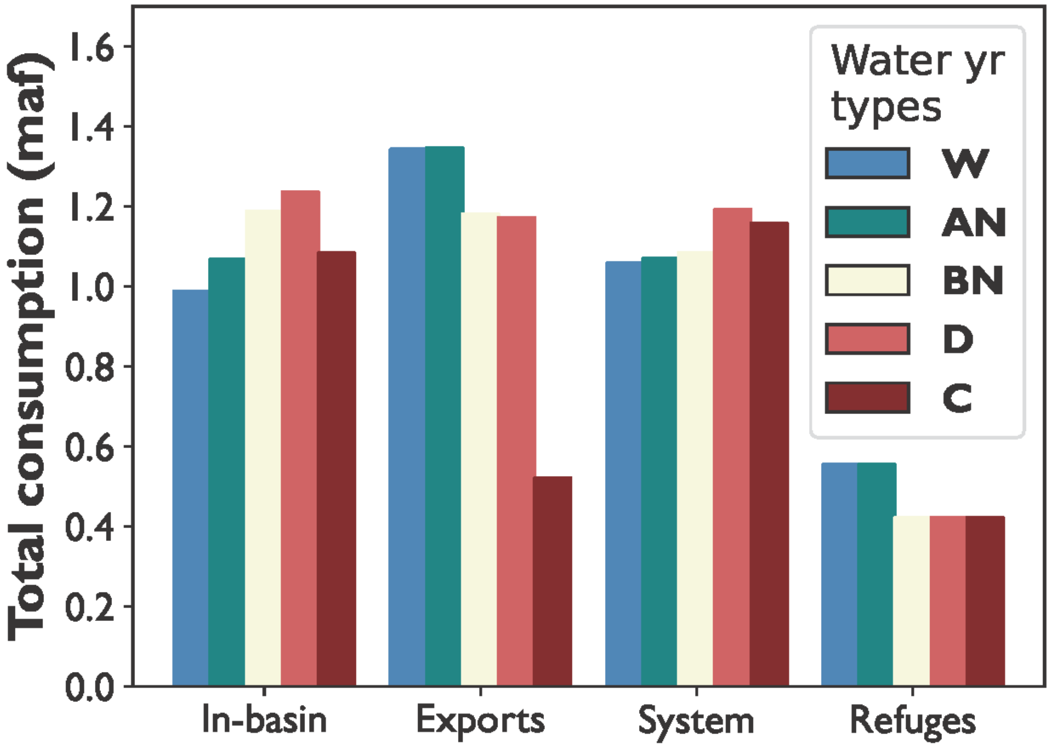

Supplemental Figure 3. Total annual water demands by water year type. W = wet years, AN = above normal years, BN = below normal years, D = dry years, and C = critically dry years using the Sacramento River Index.

Supplemental Table 1. Freshwater ecosystem goals, criteria, and timing. Mm^3/mo is millions of cubic meters per month and Tw = water temperature, juveniles include all life stages, and flow shaping volumes are in addition to environmental baseflows.

Category	Goal	Criteria	Timing	References
Flow shaping	Spring recession to flush fine sediment and cue out-migrating juvenile salmon of all runs to avoid Delta mortality	Flow >= 122.3 Mm^3/mo	Apr – June	(Yarnell et al. 2015; Nobriga et al. 2021)
Flow shaping	Winter pulse to cue downstream movement of winter-run juveniles and inundate off-channel habitat used by all runs.	Flow >= 161.2 Mm^3/mo	Jan – Mar	(del Rosario et al. 2013; Yarnell et al. 2015; Sturrock et al. 2020)
Flow shaping	Fall pulse to flush fine sediment from fall- and late fall-run spawning gravels. Cue downstream movement of winter-run juveniles and upstream migration of fall-run adults.	Flow >= 30.6 Mm^3/mo	Oct – Nov	(Yarnell et al. 2015)
Temperature control	Reduce winter-run egg and fry mortality	T_w <= 11.5 °C	June – Dec	(Zarri et al. 2019)
Temperature control	Reduce mortality of winter-run, fall-run, spring-run, and late fall-run juveniles during rearing and out-migration	T_w <= 15 °C	Year-round	(Yoshiyama et al. 1998; Richter and Kolmes 2005)
Temperature control	Reduce pre-spawn mortality in winter-run, fall-run, and late fall-run adult spawners	T_w <= 12.8 °C	June – Apr	(Yoshiyama et al. 1998; Richter and Kolmes 2005)

Supplemental Table 2. Model runs with inflow percentage, average annual inflow volume, storage capacity percentage, storage volume, and minimum storage to preserve the cold-water pool (Bm^3 is billions of cubic meters).

Environmental Assets	Inflow, %	Average Annual Inflow Volume, Bm^3	Storage Capacity, %	Storage Volume, Bm^3	Minimum Storage for Cold-water Pool, Bm^3
Percentage of inflow (pass-through)	10%<br>20%<br>30%<br>40%	0.68<br>1.35<br>20.3<br>2.71	-	-	0
Percentage of inflow (pass-through),<br>1.54 Bm^3 minimum reservoir storage	10%<br>20%<br>30%<br>40%	0.68<br>1.35<br>20.3<br>2.71	-	-	1.54
Percentage of inflow,<br>Percentage of storage capacity	10%<br>20%<br>30%<br>40%	0.68<br>1.35<br>20.3<br>2.71	10%<br>20%<br>30%<br>40%	0.56<br>1.12<br>1.68<br>2.24	0
Percentage of inflow,<br>Percentage of storage capacity,<br>1.54 Bm^3 minimum reservoir storage	10%<br>20%<br>30%<br>40%	0.68<br>1.35<br>20.3<br>2.71	10%<br>20%<br>30%<br>40%	0.56<br>1.12<br>1.68<br>2.24	1.54
